# UV-induced local immunosuppression in the tumour microenvironment of eccrine porocarcinoma and poroma

**DOI:** 10.1038/s41598-022-09490-5

**Published:** 2022-04-01

**Authors:** Maya Puttonen, Jorma Isola, Onni Ylinen, Tom Böhling, Virve Koljonen, Harri Sihto

**Affiliations:** 1 Department of Pathology, University of Helsinki, P.O. Box 63, 00014 Helsinki, Finland; 2 Faculty of Medicine and Health Technology, Tampere University, Tampere, Finland; 3 Jilab Inc, Tampere, Finland; 4 Department of Plastic Surgery, University of Helsinki and Helsinki University Hospital, Helsinki, Finland

**Keywords:** Skin diseases, Skin cancer, Cancer, Cancer microenvironment, Skin cancer, Tumour immunology

## Abstract

Eccrine porocarcinoma (EPC) is a rare malignant adnexal tumour of the skin. Part of EPCs develop from their benign counterpart, poroma (EP), with chronic light exposure and immunosuppression hypothesized to play a role in the malignant transformation. However, the impact of chronic light exposure on the microenvironment of EPCs and EPs has not been investigated yet. Although the clinical relevance of tumour infiltrating lymphocytes (TILs) and tertiary lymphoid structures (TLSs) has been established in various tumours, their distribution and significance in EPCs and EPs is still poorly understood. We characterized the distribution of TILs and TLSs using CD3, CD4, CD8, CD20 immunohistochemistry in a cohort of 10 EPCs and 49 EPs. We then classified our samples using solar-elastosis grading, analyzing the influence of ultraviolet (UV) damage on TIL density. A negative correlation between UV damage and TIL density was observed (CD4 r = −0.286, p = 0.04. CD8 r = −0.305, p = 0.033). No significant difference in TIL density was found between EPCs and EPs. TLS was scarse with the presence rate 10% in EPCs and 8.3% in EPs. The results suggest that UV has an immunosuppressive effect on the microenvironment of EPCs and EPs.

## Introduction

Eccrine porocarcinoma (EPC) is a rare malignant adnexal tumour of the skin derived from the eccrine gland. EPC affects predominantly elderly people between 60 and 80 years of age^1–5^. The most common locations are the head and neck and the lower extremities^6^. EPC etiology is still unknown but immunodeficiency has been suggested as a risk factor^7^. A portion of EPCs develop from their benign counterpart, eccrine poroma (EP)^6,8,9^, with chronic light exposure and immunosuppression hypothesized to be involved in the malignant transformation^1^. First described in 1963^10^, EPC has been considered to be an aggressive skin tumour with a high probability of recurrence^6,11,12^. Recent studies have revealed its low mortality rate and called the general belief of its aggressiveness into question^2,5,13^. However, the mortality rate is high in cases with metastatic dissemination^6,14^.

The development of genetic mutations during oncogenesis leads to the expression of tumour antigens, which trigger antitumour immune responses including the infiltration of lymphocytes into the tumour tissue^15^. Accumulating evidence from both animal models and human studies have deepened our understanding of the multifaceted role of tumour infiltrating lymphocytes (TILs) in the tumour microenvironment. TILs are associated with positive prognosis in various tumour types including skin tumours, such as Merkel cell carcinoma^16,17^ and melanoma^18^.

Secondary lymphoid organs, such as lymph nodes and spleen, are involved in antitumour immune responses^19^, as are ectopic lymphoid organs called tertiary lymphoid structures (TLSs) that develop in chronically inflamed non-lymphoid tissues, including tumours^20^. A high density of TLSs correlated strongly with a high infiltration of CD8+ T cells and CD4+ T cells in non-small cell lung cancer, which suggests that TLSs may play a key role in shaping the immunological character of the tumour microenvironment^21^. TLSs are an independent factor associated with positive prognosis in different tumours such as gastric cancer^22,23^, pancreatic ductal carcinoma^24^, and for the skin tumours Merkel cell carcinoma^25^ and melanoma^26^.

Regarding EPC and EP, there is so far one study investigating TILs in EPCs, where the degree of lymphocyte infiltration in tumours was categorized into brisk, non-brisk and absent^3^. Category was represented, but no statistically significant association between TILs and patient outcome was observed^3^. However, no comparative study of TIL distribution between EPCs and EPs has been conducted nor an investigation of TLSs in EPCs and EPs. In the current study, we performed CD3, CD4, CD8, CD20 immunohistochemistry on EPC and EP samples to feature the distribution of TILs and TLSs. In addition to this, we investigated the effects of chronic light exposure on the immunological microenvironment of EPCs and EPs.

## Methods and materials

With the approval of the Institutional Ethics Committee of Helsinki University Central Hospital [HUS/358/2018], Biobank of Northern Finland Borealis [BB_2018_2014] and Biobank of Tampere [BB2018-006], all the FFPE samples available (10 EPCs and 49 EPs) were collected from all Finnish biobanks under the coordination of the Helsinki biobank. All methods were carried out in accordance with the regulations of the biobanks. The diagnoses of EPs and EPCs were confirmed by an experienced pathologist (TB).

### Immunohistochemistry

EPC and EP FFPE samples were sectioned into 4um slices and placed on slides. This was followed by deparaffinization with xylene, dehydration with graded ethanol and incubation in 3% hydrogen peroxide for 30 min. Heat-induced epitope retrieval was carried out in sodium citrate for CD3, CD4, CD20 in 95 °C for 15 min, and for CD8 in Tris/EDTA in 95 °C for 10 min. For CD8, blocking with 2.5% goat-serum at room temperature for 15 min was performed just before application of the CD8 primary antibody. The slides were first incubated with the primary antibodies diluted in Draco Antibody Diluent (AD500) and then with the secondary antibodies for 60 min; for CD20 staining BrightVision poly HRP-Anti-Mouse IgG, ImmunologicVWR international was used, and for CD3, CD4 and CD8, rabbit HBP, Orion detection system. The primary antibodies and the incubation time are listed in Table 1. Spleen and lymph node tissues served as positive controls. Expressions were detected using a DAB Peroxidase Substrate Kit (SK-4105, Vector Laboratories; 5 min at room temperature). The slides were counterstained with hematoxylin. The slides were scanned at Jilab inc., Tampere, Finland as previously described^27^. All the tumour areas were chosen for analysis from the scanned slides. Positive cells were counted automatically by using proprietary Auto-IEL software^27,28^. Due to the occasional staining background, which prevented automatic counting, CD8 positive cells were counted manually.

**Table 1:** Primary antibodies and incubation time.

Target protein	Clone	Dilution	Incubation time and temperature	Manufacturer
CD3	SP7	1:200	30 min at RT	Thermo Fisher Scientific, Cheshire, UK
CD4	EPR6855	1:500	60 min at RT	Abcam, Cambridge, UK
CD8	EP1150Y	1:2000	30 min at RT	Abcam, Cambridge, UK
CD20	L26	1:100	60 min at RT	Abcam, Cambridge, UK

### Hematoxylin and eosin (H&E) staining and solar elastosis grading

H&E staining was performed at the laboratory of Helsinki University Hospital, Department of Pathology, according to the routine protocol. The degree of cumulative solar damage (CSD) of the surrounding skin was measured using solar elastosis grading. Solar elastosis is an accumulation of abnormal elastic tissues in the dermis caused by chronic sun exposure^29^. The grading is used clinically in melanoma diagnosis to determine if the legion is low-CSD melanoma or high-CSD melanoma^29^. Solar elastosis grades were given by using the H&E stained slides of the same tumours. The classification system has been previously described by Landi et al.^30^. In short, grade 0 was given when there was no elastic fibre in the nearby normal skin. Grade 1 samples had single elastic fibres, grade 2 samples had bunches of fibres, while grade 3 was given when there was monotonous basophilic material that had already lost its fibrillary texture^30^.

### Statistical analyses

The association between the non-continuous parameters, such as the solar elastosis grade, and the continuous parameters, such as lymphocyte densities, was evaluated using the Mann–Whitney U test. The correlation assessments of continuous distributions were carried out using the Pearson correlation test. The association between the non-continuous parameters, such as gender, EPC/EP, or location of the tumour, was evaluated using the Pearson Chi-Square test or the Fisher´s test as appropriate. All statistical analyses were performed with SPSS software (IBM SPSS Statistics for Windows ver.26). P values less than 0.05 were considered statistically significant.

## Results

### Patient and tumour characteristics

Patient and tumour data are summarized in Table 2. We established no association between EPC/ EP and gender (p > 0.05), EPC/ EP and tumour location (p > 0.05), tumour size and EPC/EP (p > 0.05). Two of the EPCs were already recurrences from the former excision and two were systemically metastasized at the time of surgery.

**Table 2:** Summary of patient and tumour data.

	EPC	EP
N	10	49
Mean age (years)	69.6 [range 22–88]	59.4 [range 7–89]
Gender (M/F/no data)	5/5	20/26/3
**Location**		
Head and neck	3	11
Trunk	4	13
Extremities	3	23
No data	–	2
Mean tumour size* (mm)	50.1 [range 5–150]	16.51 [range 3–55]
No data	1	12

*Longest diameter of the tumour.

### Lymphocyte density in EPCs and EPs

**Figure 1:**
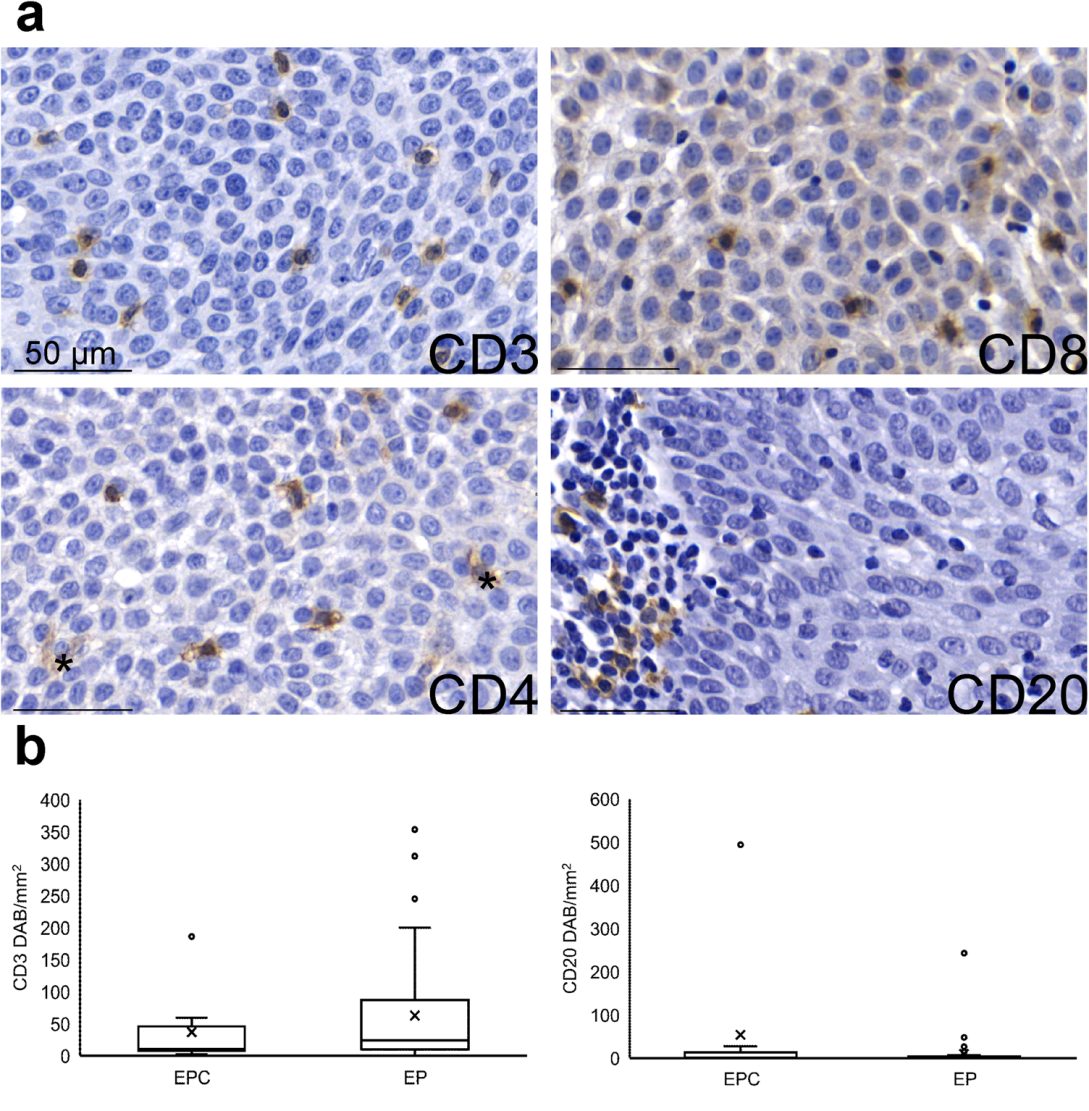
TIL density in EPCs and EPs. (**a**) Representative pictures of immunohistochemistry. * = macrophages. Scale bar = 50um. (**b**) Comparison of the density of CD3+ cells and CD20+ cells between EPCs and EPs. EPC n = 10, EP n = 48. Mean and median of the density of CD3+ cells was 36.9 ± 17.6 and 10.1 respectively in EPCs and 62.7 ± 12.0, 23.8 in EPs. p = 0.313. Mean and median of the density of CD20+ cells was 54.0 ± 48.9, 1.4 in EPCs and 9.6 ± 5.2, 1.3 in EPs.

The representative immunohistochemical staining examples are shown in Fig. 1. One EP sample was excluded from further analysis as there was no remaining tumour in the immunostained sections. Due to lack of tissue samples or a high background staining in the CD8 staining, one CD4 staining in EP, four CD8 stainings in EP and EPC were not available. The numbers of CD3+, CD4+, CD20+ lymphocytes counted automatically and the CD8+ lymphocytes counted manually are listed in Table 3.

**Table 3:** Density of each lymphocyte type (DAB+ cells/mm^2^).

	Mean	Median	Range	N
**EPC**				
CD3+	36.9 ± 17.6	10.2	1.9–186.2	10
CD4+	110.7 ± 36.6	69.4	9.2–331.1	10
CD8+	27.4 ± 10.6	13.4	1.5–75.5	8
CD20+	54.0 ± 48.9	1.5	0.0–493.8	10
**EP**				
CD3+	62.7 ± 12.0	23.8	0.0–353.4	48
CD4+	64.8 ± 14.1	34.4	0.0–489.6	47
CD8+	13.1 ± 2.2	8.6	0.0–59.0	46
CD20+	9.6 ± 5.2	1.3	0.0–242.9	48

### Distribution of TLSs

**Figure 2:**
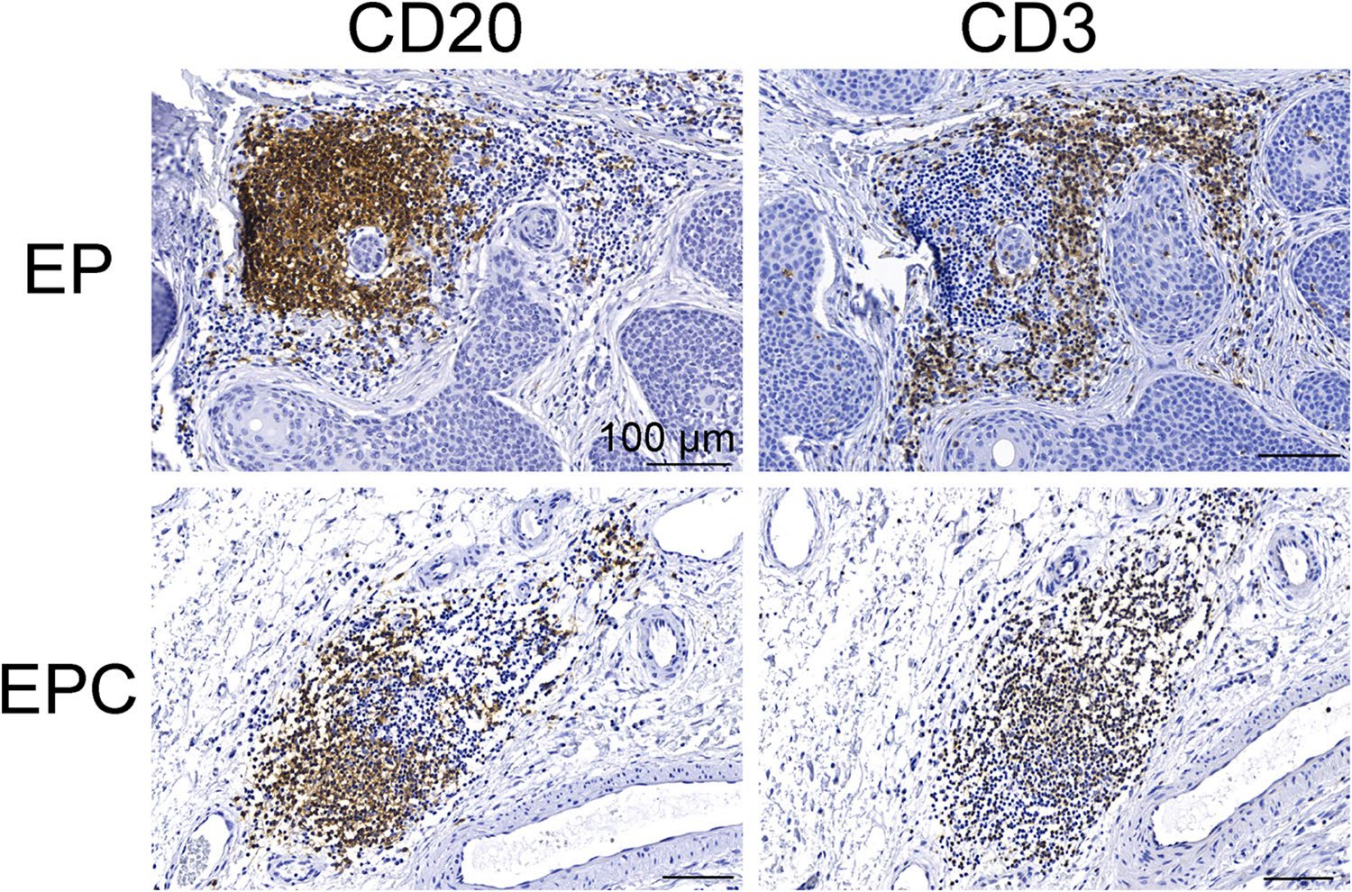
TLSs in EPCs and EPs. Dense CD20+ B cell follicle abuts a CD3+ T cell zone. Scale bar = 100um.

CD3 and CD20 immunostained parallel sections of the same tumours were evaluated for TLS. A TLS consists of a dense CD20+ B cell follicle adjacent to a CD3+ cell zone^20^. Under these criteria, 1 EPC and 4 EPs were positive for TLSs, which is 10% in EPCs and 8.3% in EPs. The representative pictures of TLSs in our samples are shown in Fig. 2.

### Relation of UV exposure to lymphocyte densities

**Figure 3:**
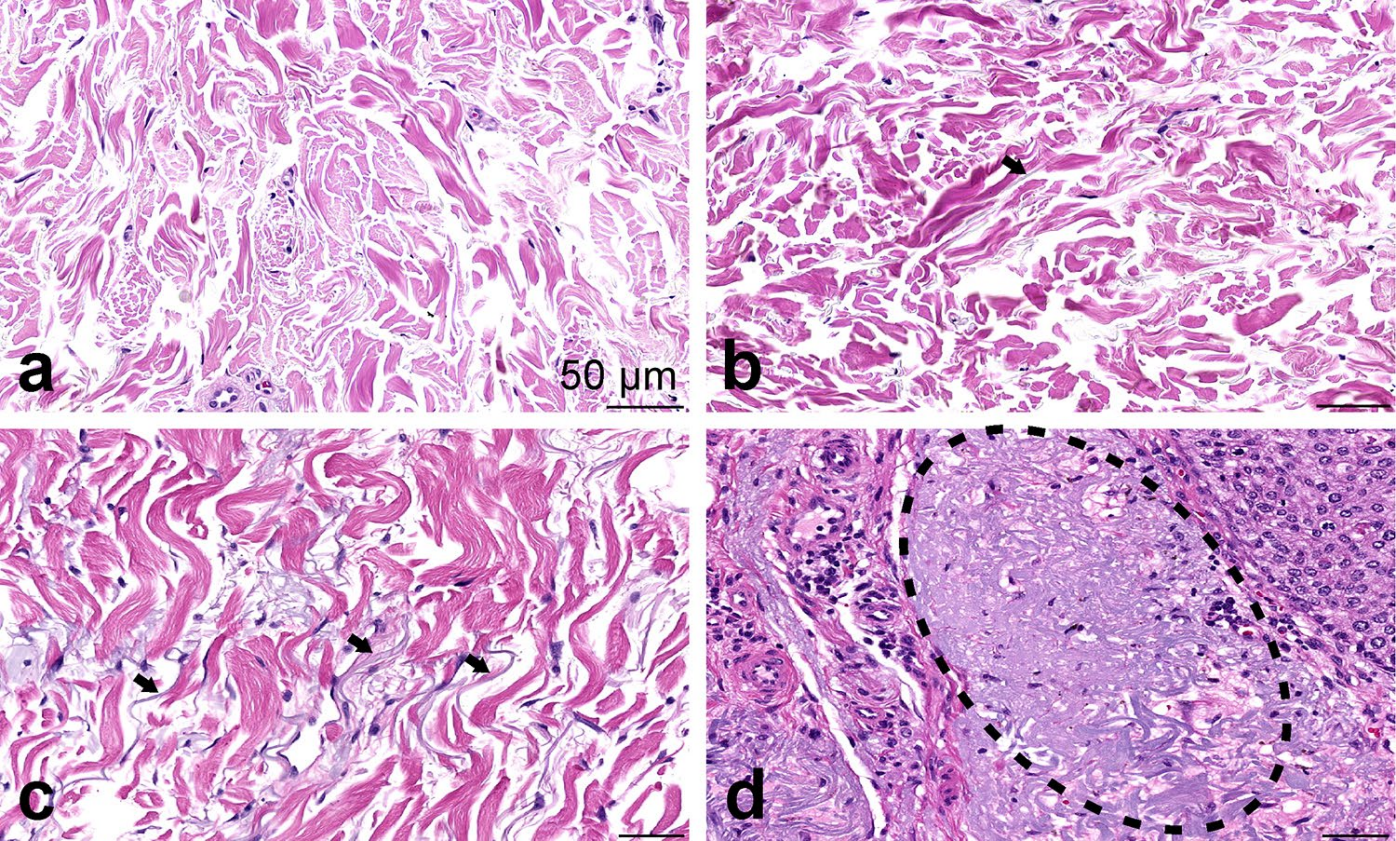
Examples of solar elastosis grading. (**a**) No visible fibres, grade 0. (**b)** Arrow points to a single elastic fibre. Grade 1. (**c**) Arrows point to bunches of fibres. Grade 2. (**d**) Dotted line circles the homogeneous basophilic material. Grade 3.

**Figure 4:**
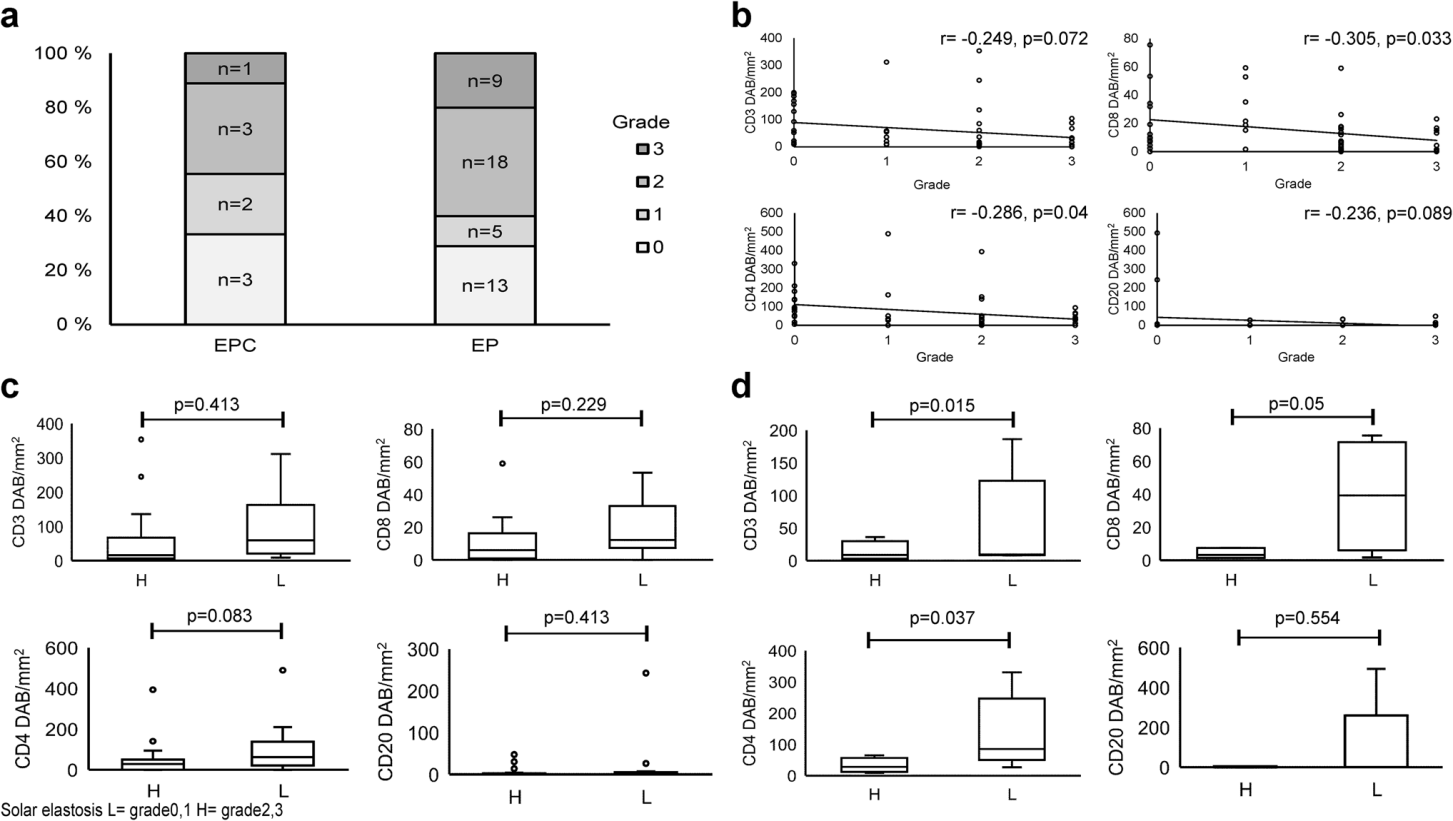
Relation of UV exposure to lymphocyte densities. (**a**) Number of samples classified into each solar-elastosis grade. The H&E staining of one EPC and four EPs did not contain adequate normal skin for the evaluation. (**b**) Distribution of the lymphocyte densities in samples of each grade. (**c)** Solar elastosis and lymphocyte density in EPCs. Sample of solar elastosis grades 0, 1 were in L (Low-solar elastosis) group and grade 2, 3 samples were in H (High-solar elastosis) group. CD3 H n = 4, L n = 5. H mean 13.9 ± 7.7, median 8.7. L: mean 54.4 ± 34.4, median 9.5. p = 0.413. CD8 H n = 3, L n = 4. H: mean 4.1 ± 1.8, median 3.3. L: mean 39.0 ± 17.2, median 39.2. p = 0.229. CD4 H n = 4, L n = 5. H: mean 32.6 ± 11.9, median 28.1. L: mean 136.0 ± 53.4, median 85.4. p = 0.063. CD20 H n = 4, L n = 5. H: mean 1.9 ± 1.3, median 1.1. L: mean 104.7 ± 97.4, median 0.89. p = 0.413. (**d**) Solar elastosis and lymphocyte density in EPs. CD3 H n = 27, L n = 17. H mean 50.4 ± 15.7, median 16.4. L: mean 93.8 ± 21.0, median 59.8. p = 0.015. CD8 H n = 25, L n = 17. H: mean 10.4 ± 2.6, median 6.0. L: mean 19.1 ± 4.1, median 12.3. p = 0.05. CD4 H n = 27, L n = 16. H: mean 47.9 ± 15.5, median 28.0. L: mean 101.3 ± 30.4, median 62.4. p = 0.037. CD20 H n = 27, L n = 17. H: mean 5.7 ± 2.3, median 1.1. L: mean 17.8 ± 14.1, median 2.0. p = 0.554.

Examples of solar elastosis in grades 0–3 are provided in Fig. 3. The number of samples classified into each group is given in Fig. 4a. The solar elastosis grade did not associate with EPC or EP , gender, tumour location or tumour size (all p-values > 0.05). Figure 4b shows the distribution of the lymphocyte densities in the samples of each grade. Statistical analysis of the association between the solar elastosis grade and the density of CD4+ and CD8+ lymphocytes revealed a significant negative correlation (CD4+ n = 52, r = −0.286, p = 0.04. CD8+ n = 49, r = −0.305, p = 0.033). The results of the same analysis conducted on EPCs and EPs separately are given as Supplementary Fig. 1.

In an attempt to explore the relation between the solar elastosis grade and the density of lymphocytes separately for EPCs and EPs despite the small sample size of EPC, the samples of grade 0 and 1 were grouped into a low-solar elastosis grade group and those of grade 2 and 3 were placed in a high-solar elastosis grade group. Plots of EPCs and EPs, given as Fig. 4c, d, respectively, showed a tendency towards a higher number of lymphocytes in samples with a low solar elastosis grade. The statistical significance of this tendency is proved in part, in that CD3+, CD8+, CD4+ TILs were more densely distributed in EPs of the low-solar elastosis group (p = 0.015, p = 0.05, p = 0.037, respectively). However, in EPCs this observation did not reach statistical significance.

## Discussion

To our knowledge, this is the first study characterizing the distribution of TILs and TLSs in both EPCs and EPs. The TIL infiltration pattern in our cohort ranged from 0 DAB+/mm^2^ to highly positive, a few hundred DAB+/mm^2^ as in density, which is in accordance with the previous report on EPCs^3^ and other skin tumours^17,31^. There was no statistically significant difference in the density of TILs between EPs and EPCs. The lack of such a difference might reflect a similar immune environment in both EP and EPC. The differences in neoantigen load and immunogenicity between EP and EPC have not been previously studied. It is possible that immunosuppression is a factor in EP development and transformation to EPC is driven by other factors such as accumulation of genomic aberrations during the cancer progression. A sequencing study reported that 36% of EPCs harboured RB1 mutations and 31% of EPCs harboured TP53 mutations while none of EPs had a mutation in the genes^32^. KRAS was recurrently mutated in EPCs, whereas not in EPs^33^.

Our series showed 10% positivity of TLSs in EPCs and 8.3% in EPs. Our figures are extremely low compared to most of the other tumours reported so far. For instance, in a cohort of 125 breast cancer samples, 60% were TLS positive^34^. In a cohort of 351 colorectal cancer samples, 78.6% were TLS positive^35^ and all the 534 pancreatic ductal carcinomas were TLS positive^24^. However, in clear cell renal cell carcinomas, TLSs were scarce^36^ as in our EPCs and EPs.

In this study, we found that the more UV-induced damage observed in the skin near the tumour, the less CD3+, CD4+ and CD8+ TILs were identified. One hypothesis of a cause to this phenomenon is UV-induced immunosuppression. UV-damage leads to induction and activation of immunosuppressive regulatory T cells, decreased number and function of Langerhans cells^37^ and increased release of immunosuppressive mediators, such as interleukin (IL)-10^38^. Langerhans cells function as antigen-presenting cells in the skin and initiate an immunological response by interacting with lymphocytes, thus affecting negatively to Langerhans cell function interferes with the immune system in the skin^39^. It is also known that in phototherapy used for dermatological diseases, irradiation of UV-B and UV-A plus psoralen causes down-regulation of the IL-23/ T-helper 17 (Th17) cell axis and induces Tregs involving CTLA4 signaling^40^. UV-damage also decreases the number of dendritic epidermal T cells (DETCs)^41^, which are known to induce CD8+ T cells^42^. Additionally, UVB radiation has been proven to induce apoptosis of cutaneous T-cells in both in vitro and in vivo settings^39^.

On the other hand, exposure to UV could result in an increased number of TILs. For instance, a cohort of Merkel cell polyoma virus negative Merkel cell carcinomas exhibited high TILs and a high PD-L1 expression corresponding with a higher UV-associated mutation burden^43^. Our study suggests that in the microenvironment of EPs and EPCs, the influence of UV-induced immunosuppression exceeds the TIL-induction reacting to mutations. One of the possible mechanisms under this observation is immune evasion caused by mutations in genes coding components of class I MHC. Although there is no study so far investigating this topic in EPs and EPCs, studies with colorectal cancers have revealed that in tumours with high microsatellite-instability, the expression of *HLA-A*, *HLA-B*, and *HLA-C* were decreased and incidence of mutations in *B2M* were increased^44^. *HLA-A*, *HLA-B*, and *HLA-C* encode the alpha chain of class I MHC and *B2M* encodes the beta chain of class I MHC, both enabling the peptide to be presented to specific receptors on the surface of cytotoxic T cells^44^. Also in non-small cell lung cancer, a high neoantigen burden was associated with loss of heterozygosity in HLA^45^. These immune evasion by tumours with high mutation burden are hypothesized to be the result of gene mutations that indirectly decrease the expression of the genes^44^. Additionally, mutations resulting in upregulation of WNT signalling have been proven to be associated with lower T-cell density in colorectal cancer^46^ and in melanoma^47^. According to a study of non-small cell lung cancer, treatment with EGFR tyrosine kinase inhibitors increased tumour mutation burden and decreased CD8+ TIL densities^48^. Clarification of the association between tumour mutation burden and TIL infiltration in EPs and EPCs warrants further genomic investigations.

The mean number of CD4 cells was higher than that of CD3 cells. This could be due to the macrophage population, which also stains positive with CD4 marker. Although we calibrated the parameters of the counting system, it was not possible to completely prevent macrophages from being counted as positive. The strong correlation of the CD4+ density with the CD3+ density (r = 0.754, p = 0.000. data not shown) supports the hypothesis that even though part of macrophages were counted, this happened evenly throughout the samples, justifying the use of a number of CD4+ cells independently from that of other TIL types in the analysis. In this study, the number of samples was limited because of the rarity of EPC. This weakened the power of statistical analyses. Also, as most of the subjects lived till the end of follow-up, the prognostic or predictive value of TIL could not be analyzed in this study. This topic warrants further investigation, preferably with a bigger cohort.

Immunosuppression has been suggested as one of the risk factors of EPC and of malignant transformation from EP to EPC^1^. An altered immunological environment could be one of the mechanisms underlying the contribution of UV radiation to carcinogenesis.

## Supplementary Information

Supplementary Legends.

Supplementary Figure S1.

## Data Availability

The datasets generated during and/or analysed during the current study are available from the corresponding author on reasonable request. However, raw datasets containing identifying information on individual patients are confidential under Finnish law and cannot be made available as such.
