# Supplementary material for: UV-induced local immunosuppression in the tumour microenvironment of eccrine porocarcinoma and poroma
Source: Sci Rep. 2022 Apr 1;12:5529. doi: 10.1038/s41598-022-09490-5 (PMC8976087; doi:10.1038/s41598-022-09490-5)
Supplement: Supplementary file 1 — Supplementary Legends. [file 41598_2022_9490_MOESM1_ESM.docx]

Supplementary Fig.1. Relation of UV exposure to lymphocyte densities. a. Distribution of the lymphocyte densities in EPC samples of each grade. b. Distribution of the lymphocyte densities in EP samples of each grade.
