# Supplementary figures and images for: UV-induced local immunosuppression in the tumour microenvironment of eccrine porocarcinoma and poroma
Source: Sci Rep. 2022 Apr 1;12:5529. doi: 10.1038/s41598-022-09490-5 (PMC8976087; doi:10.1038/s41598-022-09490-5)

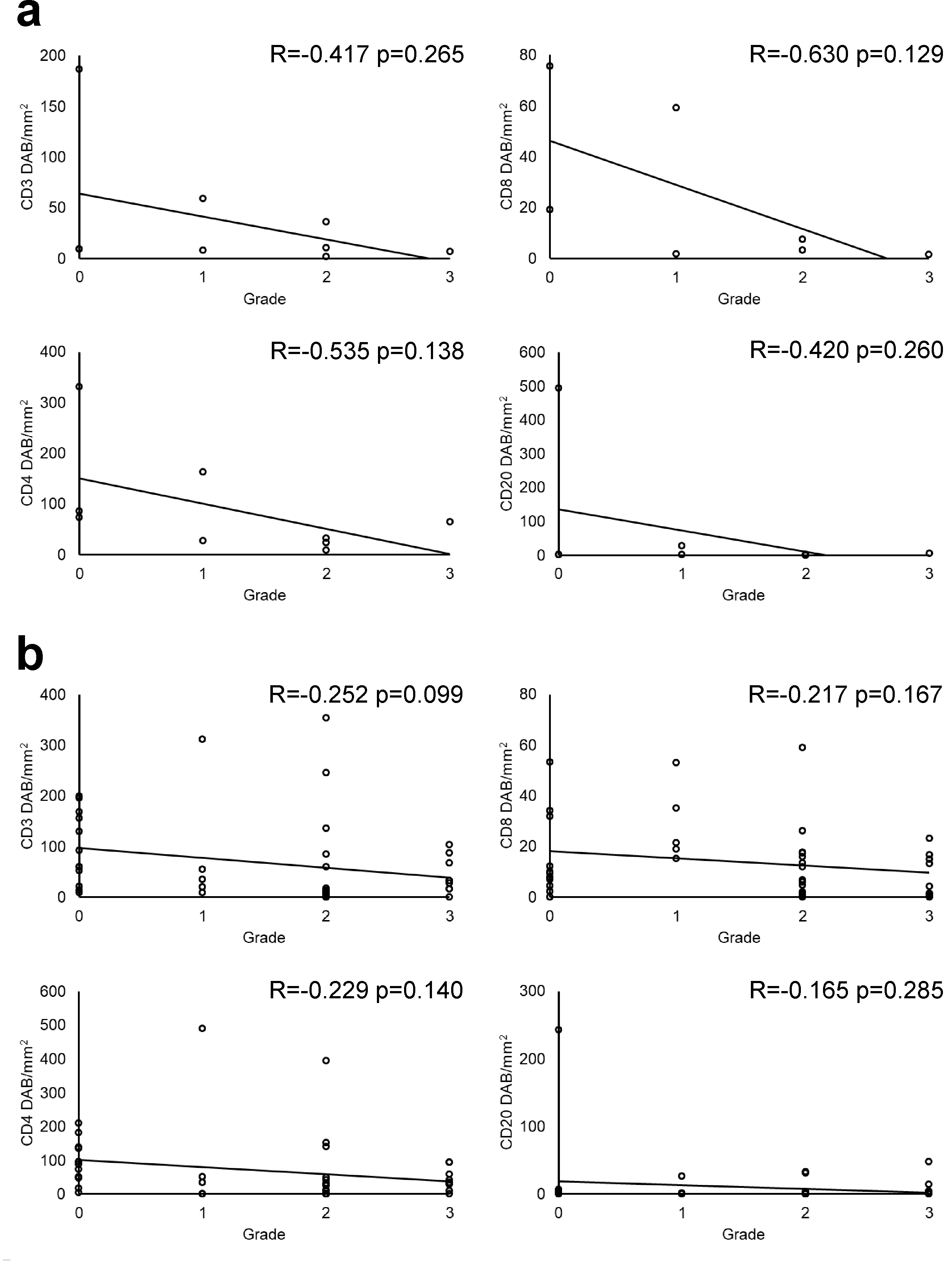

Supplement: Supplementary file 2 — Supplementary Figure S1. [file 41598_2022_9490_MOESM2_ESM.tif]
